# Comparison of Functional Outcomes and Its Association With the Risk Factors in Patients With Anterior and Posterior Circulation Ischemic Stroke

**DOI:** 10.7759/cureus.99637

**Published:** 2025-12-19

**Authors:** Roksana Yasmin, Mohammad Bahadur Ali Miah, Md. Shahidullah, Md. Moniruzzan Bhuiyan, Mehedi Hasan, SK Mahbub Alam, Subash Kanti Dey, Md Masud Rana, Muhammad Rezeul Huq

**Affiliations:** 1 Neurology, Bangladesh Medical University, Dhaka, BGD; 2 Neurology, Combined Military Hospital, Dhaka, BGD

**Keywords:** anterior circulation ischemic stroke, functional outcome, ischemic stroke, posterior circulation ischemic stroke, risk factors

## Abstract

Background: Stroke is a highly debilitating condition resulting in significant morbidity and mortality on a global scale. Anterior circulation ischemic stroke (ACIS) and posterior circulation ischemic stroke (PCIS) are distinguished by varying risk factors and functional outcomes. This study is designed to identify the disparities in functional outcomes and their association with risk factors in ACIS and PCIS patients, as assessed by the modified Rankin Scale (mRS) at day 90 post-onset of stroke symptoms.

Materials and methods: This prospective, observational, comparative study was conducted at the Department of Neurology at Bangladesh Medical University (formerly Bangabandhu Sheikh Mujib Medical University [BSMMU]), Dhaka, over 16 months, from May 2023 to September 2024. A total of 116 ischemic stroke patients (58 in the ACIS group and 58 in the PCIS group) were included in this study. A comprehensive medical history and thorough clinical examinations were performed, along with relevant investigations to assess the risk factors. The functional outcomes of the patients were evaluated using mRS by the principal investigator on day 90.

Results: The mean (±SD) age was 59.0 (±12.50) years in the ACIS group and 58.9 (±15.7) years in the PCIS group; this difference was not statistically significant (p = 0.969). ACIS showed a higher prevalence of cardiac illness (p = 0.002), while hypertension (p = 0.026) and smoking (p = 0.007) were notable in PCIS. The study revealed that patients with PCIS exhibited poorer outcomes in comparison to the ACIS group. Among PCIS patients, 32 (55.2%) had poor outcomes (mRS 3-6), whereas among ACIS patients, 15 (25.9%) did; this difference was statistically significant (p < 0.001). Furthermore, ACIS patients were more likely to experience mortality (8.6% vs. 1.7%). Hypertension was associated with a poor functional outcome in both groups (p = 0.023 for ACIS and p = 0.002 for PCIS patients). In contrast, diabetes mellitus and obesity were associated with a worse functional outcome in ACIS and PCIS patients, respectively (p < 0.001 and p = 0.021).

Conclusion: Patients with PCIS had a higher prevalence of modifiable risk factors like hypertension and smoking, and showed significantly poorer functional outcomes than those with ACIS at 90 days. Additionally, higher frequencies of cardiac illness and mortality were notable in ACIS. These study findings will help clinicians to plan for specific prevention, acute management, and rehabilitation of Bangladeshi stroke patients.

## Introduction

Stroke is the second leading cause of death [1] and the third leading cause of severe disability in adults worldwide, with 80-87% of strokes being ischemic [2-4]. According to the Global Burden of Disease Study, more than 12 million new strokes occur annually, resulting in over 6 million deaths and substantial long-term disability. The mortality and morbidity were higher in low-income countries, possibly due to limited access to preventive care or management of acute stroke [5].

Ischemic stroke occurs due to the obstruction of blood vessels, which restricts blood supply, resulting in cerebral infarction and associated neurological symptoms corresponding to the affected part of the brain. The underlying pathophysiological mechanisms include large-artery atherosclerosis, cardioembolism, small-vessel occlusion (lacunar stroke), and other less common causes such as arterial dissection or hypercoagulable states [6]. The size of the infarct depends on the duration and severity of ischemia, collateral circulation, and the metabolic demands of affected brain tissue.

Ischemic stroke can be further specified according to its vascular territory as anterior circulation ischemic stroke (ACIS, from internal carotid arteries) and posterior circulation ischemic stroke (PCIS, from vertebrobasilar arteries and their branches) [7,8]. The anterior circulation supplies the cerebral hemispheres, including the frontal, parietal, and lateral temporal lobes. In contrast, the posterior circulation nourishes the brainstem, cerebellum, occipital lobes, and thalami. This vascular distinction gives rise to different pathophysiological patterns and clinical presentations. Approximately 20% of ischemic strokes involve the posterior circulation territory [9,10]. The posterior circulation has some unique features, like two large arteries that unite to make another large artery. The arteries are tortuous with higher shear stress, making them vulnerable to atherosclerotic changes [11].

Notable modifiable risk factors for ischemic stroke in the Bangladeshi population are hypertension (57.6%), smoking (44.6%), tobacco use (24.3%), oral contraceptive pill (OCP) usage in females (40% of female stroke cases), diabetes (23%), ischemic heart disease (17.1%), obesity (10.6%), and dyslipidemia (5.3%) [12]. The risk factors are similar in both anterior and posterior circulation strokes, except that cardiac diseases like atrial fibrillation and heart valve disease are more commonly associated with ACIS [13]. Other studies have indicated that diabetes mellitus, hypertension, and smoking are more common in posterior circulation stroke [14,15].

These variations in vascular anatomy, pathophysiology, and risk factors may translate into differences in clinical outcome and recovery potential between ACIS and PCIS [13-16]. Treatment might differ in the acute and chronic phases of ACIS and PCIS. As clinical features are different and vague, like decreased consciousness or vertebrobasilar symptoms rather than typical hemispheric syndromes, PCIS patients usually get a lower percentage of thrombolysis compared to ACIS [17]. PCIS strokes are often lacunar, whereas ACIS strokes are more cardioembolic, so the overall management approach may differ [17]. Some studies report a lower mortality rate in PCIS [18], whereas others describe poorer outcomes due to atypical presentation, delayed diagnosis, and underutilization of reperfusion therapies [19].

Despite these observations, few studies have systematically compared risk factors, pathophysiology, and functional outcomes between ACIS and PCIS, particularly in South Asian populations. Although ischemic stroke is a common and well-studied condition, most evidence comes from large national or international cohorts where subgroup differences may be diluted by sample heterogeneity. Less is known about how specific clinical or demographic subgroups of stroke patients differ in smaller, local populations that reflect real-world clinical practice. The available evidence remains limited and sometimes contradictory. Therefore, this study aims to compare functional outcomes and their association with risk factors among patients with anterior and posterior circulation ischemic stroke in a Bangladeshi cohort.

## Materials and methods

Study settings and population

This prospective comparative study was carried out from May 2023 to September 2024. The study was conducted in the Stroke and Neuro-intervention Clinic and the Inpatient and Outpatient Department of Neurology of Bangladesh Medical University, BMU (formerly known as Bangabandhu Sheikh Mujib Medical University, BSMMU). Acute ischemic stroke patients, with a duration of up to seven days from symptom onset, were categorized into ACIS & PCIS. As PCIS stroke patients receive less thrombolysis compared to ACIS patients, to exclude the effect of thrombolysis and mechanical thrombectomy, only the patients who did not receive thrombolysis or mechanical thrombectomy due to delayed arrival or other contraindications were included in the study. Only adult patients aged 18 years or older were included in this study. Patients who were suffering from symptomatic recurrent ischemic stroke, hemorrhagic stroke, or subarachnoid hemorrhage and were unwilling to give informed consent were excluded from the study.

Study procedure

A total of 116 patients (58 in the ACIS group and 58 in the PCIS group) were selected based on specific inclusion and exclusion criteria. The sample size was calculated using the sample size calculation for two independent proportions. Resource limitations, including funding and staffing, restricted the sample size to an 80% confidence level and 70% power. In the equation, the proportion of unfavorable outcomes in patients with anterior circulation stroke was 42%, and the proportion of unfavorable outcomes in patients with posterior circulation stroke was 26% [17]. Subjects were selected by the purposive sampling method. All the patients who fulfilled the inclusion and exclusion criteria within the study period were included. Informed written consent was obtained from each patient or their attendant. The patients' demographic details were recorded. A comprehensive history, physical examination, and neurological assessment were performed while taking into account the study variables. All relevant investigations, including MRI of the brain and at least one modality of vascular imaging (MRA/CTA/DSA/carotid duplex study), were completed. The patients' functional outcomes were evaluated using the Modified Rankin Scale (mRS) [20] by the principal investigator during follow-up visits at 90 days after the index ischemic stroke (from stroke onset).

Data processing and analysis

Statistical analysis was carried out using IBM Corp. Released 2020. IBM SPSS Statistics for Windows, Version 26. Armonk, NY: IBM Corp. Continuous variables (age) were presented as mean ± standard deviation, while categorical variables were expressed as percentages (sex, risk factors, mRS). Group comparisons for continuous variables were conducted using unpaired t-tests. The categorical variables were tested using chi-squared tests, with a significance level set at p < 0.05 to determine statistical significance.

Ethical consideration

Ethical clearance from the Institutional Review Board (BSMMU/2023/8804) was obtained before the study. Informed written consents were obtained from all the participants and/or attendants. The patients and their relatives were assured about the confidentiality of all the information.

## Results

The age distributions of both groups were analogous (p = 0.969), with a substantial majority of participants (43 patients, 74.1% in the ACIS, and 41 patients, 70.7% in the PCIS) falling within the 50-79-year age range. Furthermore, 18 (31.0%) participants in both groups predominantly fell within the 60-69-year age range. The sex distribution among the research participants revealed a predominance of male patients in both groups, accounting for 35 (60.3%) in the ACIS group and 30 (51.7%) in the PCIS group. Notably, there was no statistically significant difference in age and sex distribution between the two groups (p>0.05) (Table 1).

**Table 1:** Comparison of demographic profiles of patients with anterior circulation ischemic stroke and posterior circulation ischemic stroke p-value obtained by unpaired t-test and chi-square test; p<0.05 considered as a level of significance (* significant); a = Pearson chi-square value, statistic value obtained by chi-square test; b = t value, statistic value obtained by unpaired t-test; c = p-value obtained by chi-square test; d = p-value obtained by unpaired t-test

	Anterior circulation ischemic stroke (n=58), n (%)	Posterior circulation ischemic stroke (n=58), n (%)	Statistical value	p-value
Sex				
Male	35(60.3)	30(51.7)	0.875^a^	0.350^c^
Female	23(39.7)	28(48.3)		
Age group (year)				
<30	1(1.7)	4(6.9)	5.168^a^	0.522^c^
30-39	3(5.2)	0(0.0)		
40-49	8(13.8)	9(15.5)		
50-59	13(22.4)	11(19.0)		
60-69	18(31.0)	18(31.0)		
70-79	12(20.7)	12(20.7)		
80 and above	3(5.2)	4(6.9)		
Total	58(100.0)	58(100.0)		
Mean±SD Range (min-max)	59.0±12.50 (25-90)	58.9±15.7 (19-92)	-0.039^b^	0.969^d^

ACIS showed a higher prevalence of cardiac illness (ischemic heart disease, atrial fibrillation, heart failure, valvular heart disease, etc.) (p = 0.002), while hypertension (p = 0.026) and smoking (p = 0.007) were notable in PCIS. Other factors showed no significant variance (Table 2).

**Table 2:** Association of the risk factors between two groups (N=116) A p-value obtained by the chi-square test, p<0.05, was considered as a level of significance (* significant)

Risk factors	Anterior circulation ischemic stroke (n=58), n (%)	Posterior circulation ischemic stroke (n=58), n (%)	Pearson Chi-square value	p-value
Hypertension	23 (39.7)	35 (60.3)	4.966	0.026*
Diabetes mellitus	36 (62.1)	34 (58.6)	0.144	0.704
Dyslipidemia	35 (60.3)	33 (56.9)	0.142	0.706
Obesity	25 (43.1)	18 (31.0)	1.811	0.178
Smoking	15 (25.9)	29 (50.0)	7.177	0.007*
Cardiac disease	15 (25.9)	3 (5.2)	9.469	0.002*
Family history of stroke	18 (31.0)	27 (46.6)	2.941	0.086

There was a significant difference in the 90-day outcomes for patients with ACIS and PCIS (p < 0.001). For convenience in conducting a statistical study, both patient groups were divided into good-outcome (mRS score 0-2) and poor-outcome (mRS score 3-6) groups. Patients with ACIS had a higher rate of good outcomes (mRS score 0-2) than those with PCIS. Conversely, patients with PCIS were more likely to have poor outcomes (mRS score 3-6) than those with ACIS. Mortality was higher in ACIS (8.6%) patients compared to PCIS patients (1.7%) (Table 3).

**Table 3:** Frequency of functional outcome at day 90 of stroke between two groups (N=116) p-value obtained by unpaired t-test and chi-square test; p<0.05 considered as a level of significance (* significant); a= Pearson chi-square value, statistic value obtained by chi-square test; b= t value, statistic value obtained by unpaired t-test; c= p-value obtained by chi-square test; d= p-value obtained by unpaired t-test

mRS score at day 90	Anterior circulation (n=58), n (%)	Posterior circulation (n=58), n (%)	Statistical value	p-value
No significant disability (mRS 1)	7 (12.1)	9 (15.5)	21.039^a^	<0.001^c*^
Slight disability (mRS 2)	36 (62.1)	17 (29.3)		
Moderate disability (mRS 3)	6 (10.3)	21 (36.2)		
Moderately severe disability (mRS 4)	2 (3.4)	7 (12.1)		
Severe disability (mRS 5)	2 (3.4)	3 (5.2)		
Dead (mRS 6)	5 (8.6)	1 (1.7)		
Good outcome (mRS score 0-2)	43 (74.1)	26 (44.8)	10.337^a^	0.001^c^*
Poor outcome (mRS score 3-6)	15 (25.9)	32 (55.2)		
Total	58 (100.0)	58 (100.0)		
Mean ± SD	2.28±1.19	2.90±1.24	2.746^b^	0.007^d^*

The patients of ACIS with diabetes mellitus and hypertension exhibited a higher likelihood of experiencing a poor outcome (p<0.001 and p = 0.023, respectively). Conversely, individuals with PCIS who had hypertension (p = 0.002) and obesity (p = 0.020) were associated with an elevated risk of a poor outcome (Table 4).

**Table 4:** Association of risk factors with functional outcome in two groups (N=116) p-value obtained by chi-square test; p<0.05 considered as a level of significance (* significant); ACIS = Anterior circulation ischemic stroke; PCIS = Posterior circulation ischemic stroke

Risk factors	Functional outcome by mRS score at 90 days		Statistical value	p-value
	Poor outcome (n=47) n (%)	Good outcome (n=69) n (%)		
ACIS	(n=15)	(n=43)		
Hypertension	14(93.3)	9(20.9)	24.362	<0.001*
Diabetes mellitus	13(86.7)	23(53.5)	5.200	0.023*
Dyslipidemia	9(60.0)	26(60.5)	0.001	0.975
Obesity	7(46.7)	18(41.9)	0.105	0.746
Smoking	5(33.3)	10(23.3)	0.589	0.433
Cardiac disease	5(33.3)	10(23.3)	0.589	0.443
Family history of stroke	6(40.0)	12(27.9)	0.760	0.383
PCIS	(n=32)	(n=26)		
Hypertension	25(78.1)	10(38.5)	9.431	0.002*
Diabetes mellitus	21(65.6)	13(50.0)	1.444	0.230
Dyslipidemia	18(56.3)	15(57.7)	0.012	0.912
Obesity	14(43.8)	4(15.4)	5.393	0.020*
Smoking	19(59.4)	10(38.5)	2.510	0.113
Cardiac disease	2(6.3)	1(3.8)	0.169	0.681
Family history of stroke	16(50.0)	11(42.3)	0.341	0.559

## Discussion

This prospective comparative study compared the functional outcomes in patients with these types of stroke and found significant disparities. The data analysis revealed that the majority of patients in both groups were between 60 and 69 years old (18 patients, 31% in both ACIS & PCIS groups). The mean (±SD) age was 59.0 (±12.50) years in the ACIS group and 58.9 (±15.7) years in the PCIS group. A previous study in Bangladesh found a mean age of 58.91 ± 7.03 years among stroke patients [21]. Another study in China reported mean ages of 62.71 (±18.36) years for anterior circulation infarction and 61.77 (±14.15) years for posterior circulation infarction [12], consistent with our study. Considering these findings, we can consider that age is not related to the blood supply-based ischemic stroke subtypes.

The majority of patients in both groups were male (35 patients, 60.3%, and 30 patients, 51.7%, respectively) without any statistically significant difference (p=0.349). This aligns with previous studies by Sommer et al. (2018), which showed no significant sex differences [19]. On the other hand, Zürcher et al. (2019) found that 53.5% of patients with ACIS and 61.4% with PCIS were male, showing significant male predominance [17]. Historically, it is considered that men suffer from stroke more than women. Smoking, behavioral differences like a less controlled lifestyle, or lower healthcare-seeking behavior may contribute to a greater incidence of stroke among men. However, at present, there are no sex-based differences in stroke territory.

In both groups, diabetes emerged as the most prevalent risk factor. Specifically, 36 (62.1%) patients with ACIS presented with diabetes, compared to 34 (58.6%) in the PCIS group. Notably, a study in Iran also reported a frequency of 40.8% for diabetes in ACIS and 60% in PCIS [14]. The prevalence of hypertension proved to be higher in PCIS (35 patients, 60.3%) than in the ACIS group (23 patients, 39.7%), representing a statistically significant difference (p=0.026). Furthermore, the prevalence of smoking was higher in PCIS (29 patients, 50%) than in ACIS (15 patients, 25.9%), a disparity that also demonstrated statistical significance (p=0.007). This aligns with the findings of an Iranian study [14].

Cardiac disease was significantly more frequent in ACIS patients (15 patients, 25.9% vs. 3 patients, 5.2%) (p=0.002). Several studies similarly identified a higher frequency of cardiac disease in the ACIS group [7,12,22]. However, our study did not reveal statistically significant differences in the distribution of diabetes mellitus, dyslipidemia, obesity, and family history of stroke between the two groups (p>0.05). This corroborates the findings of two Iranian studies [14,23]. Diabetes mellitus was not associated with ACIS & PCIS in another Egyptian study, also [24].

The mRS is a well-validated tool used to measure functional outcomes after a stroke. It is commonly employed in stroke trials and prospective studies of post-stroke disability. Among the patients in the PCIS, 32 (55.2%) had poor outcomes, and 26 (44.8%) had good outcomes. Patients in the ACIS group had significantly better outcomes compared to the PCIS group (P=0.001). A total of 43 (74.1%) patients with ACIS had good outcomes (independent, mRS 0-2), and 32 patients (55.2%) with PCIS had poor outcomes (death or dependency, mRS 3-6). These findings are consistent with several previous studies [19,25]. PCIS patients often have dizziness or imbalance, which may contribute to the long-term poorer outcomes. 

Another study found the adjusted clinical outcome at three months was similar in both groups [17]. However, a Swiss study found better median three-month functional outcomes in PCIS patients [7]. The diverse findings in different studies may be attributed to varying methodologies of study, sampling techniques, and various ethnicities.

The mortality was 8.6% in the ACIS group compared to 1.7% in the PCIS group. All the death patients had advanced age, multiple comorbidities, stroke due to large artery atherosclerosis, and an initial high mRS. Similar mortality rates were found in both groups of stroke in another study [26]. In the case of minor strokes, both groups had identical mortality [25]. In our study, the number of dependent persons was higher in PCIS patients, and mortality was higher in ACIS patients. However, as the sample size in our study was small, a larger-scale study is needed to confirm these findings in the Bangladeshi population.

Regarding the association of functional outcome with risk factors, this study found that poor functional outcomes of PCIS were associated with hypertension (p = 0.002) and obesity (p = 0.020). A recent study also found an association of hypertension with worse outcomes in PCIS after mechanical thrombectomy [27]. Another study revealed that in ischemic stroke patients, being overweight or obese was not associated with decreased mortality or better functional recovery, but being underweight predicted unfavorable outcomes [28]. Poor functional outcome of ACIS was associated with hypertension (p < 0.001) and diabetes (p = 0.023) in this study. A study conducted in Iran found poor functional outcomes related to diabetes and hypertension in both vascular territories [29].

Our study has several limitations. First, the sample size was relatively small and drawn from a single center, which means that the results cannot be generalized to the entire Bangladeshi population. Additionally, a 90-day follow-up period may not be sufficient to adequately compare the functional outcomes between the two stroke groups, as patients may continue to improve over time. Furthermore, we did not take into account specific potential confounders, such as socioeconomic status and access to rehabilitation services, both of which may affect patient outcomes.

We recommend conducting larger multicenter studies with extended follow-up periods, ensuring that all participants receive standard rehabilitation services.

## Conclusions

Our findings show significant differences between outcome and risk factors of ACIS & PCIS patients. People with PCIS had greater long-term disability at 90 days, while those with ACIS had a slightly higher risk of death. Hypertension and smoking were prevalent in PCIS, whereas cardiac diseases were more frequent in ACIS. These findings will help to prevent and manage different types of strokes in more systemic ways. Larger studies are needed for further confirmation of these findings and to formulate policies at the national level for an individualized approach in stroke prevention and management.
